# Supplementary material for: Patterns of Intron Gain and Loss in Fungi
Source: PLoS Biol. 2004 Nov 30;2(12):e422. doi: 10.1371/journal.pbio.0020422 (PMC532390; doi:10.1371/journal.pbio.0020422)
Supplement: Table S1 — Also available at http://genes.mit.edu/NielsenEtAl/. (4.3 MB ZIP). [file pbio.0020422.st001.zip › NielsenEtAl/html/794.html]

AN1545.1.NCU09377.1.MG05637.1.FG01369.1


```
 CLUSTAL W (1.82) Multiple Sequence Alignments - Introns Inserted


Sequence 1: NCU09377.1	481 aa
Sequence 2: MG05637.1	507 aa
Sequence 3: FG01369.1	476 aa
Sequence 4: AN1545.1	495 aa
Alignment Length: 530 aa
Number Identitical Residues: 391 aa
Alignment Score (without introns) 15810


MG05637.1 	MVEADTNSPTWKFTQ2CFGDKGDVEDITEGKHPP1DIISTVEFDHTGNYLATGDKGGRVV
NCU09377.1	MVETDTNSPTWKFTQ2CFGDKGDVEDITE----A1DIISTVEFDHTGNYLATGDKGGRVV
FG01369.1 	------MSSPLAFSK~VSSSTSYSNRYACSG-TA~DIISTVEFDHTGNYLATGDKGGRVV
AN1545.1  	MVDRDPGTPTWKFTQ2CFGDKGDVEDITE----A1DIISTVEFDHTGNYLATGDKGGRVV
          	  . .. :..  *::   ....  :  :     . *************************

MG05637.1 	LFERNET0KKTCEYKFHTEFQSHEPEFDYLKSLEIEEKINKIKWCRRQNASHYLLSTNDK
NCU09377.1	LFERNET0KKTCEYKFHTEFQSHEPEFDYLKSLEIEEKINKIKWCRRQNASHYLLSTNDK
FG01369.1 	LFERNET0KKTCEYKFHTEFQSHEPEFDYLKSLEIEEKINKIKWCRRQNASHYLLSTNDK
AN1545.1  	LFERNET0KKTCEYKFHTEFQSHEPEFDYLKSLEIEEKINKIKWCRRQNASHFLLSTNDK
          	******* ********************************************:*******

MG05637.1 	TIKLWKVFEKSLKVVAENNLSHDLTPANIAGGGGAPRPPPASTFRNAADLKLPRLTHHDT
NCU09377.1	TIKLWKVFEKSLKVVAENNLSHDLTPASVAGGGGAPRALPHTQFRSPADLKLPRLTHHDT
FG01369.1 	TIKLWKVFEKSLKVVAENNLSHDVTPGSIAGGGGAPKPLPAHQFKNAADLKLPRLTHHDT
AN1545.1  	TIKLWKVFDKSLKVVAENNLSTELTPGGVGGGG-APR-APRVSFKDSSALKLPRMTHHDT
          	********:************ ::**..:.*** **:  *   *:..: *****:*****

MG05637.1 	VVAAVPRRTYANAHAYHINSISVNSDGETFISSDDLRINLWNLNIQEQSFNIVDIKPANM
NCU09377.1	VVAAVPRRTYANAHAYHINSISVNSDGETFISSDDLRINLWNLNIQDQSFNIVDIKPANM
FG01369.1 	VVAAVPRRTYANAHAYHINSISVNSDGETFISSDDLRINLWNLNIQDQSFNIVDIKPANM
AN1545.1  	VVAAVPRRTYANAHAYHINSISVNSDGETFISSDDLRVNLWNLNIQDQSFNIVDIKPANM
          	*************************************:********:*************

MG05637.1 	EELTEVITAAEFHPASCNVFMYASSKGTIKLADMRESALCDQHAK---------------
NCU09377.1	EELTEVITAAEFHPLSCNWFMYASSKGTIKLADMRERALCDQHAK---------------
FG01369.1 	EELTEVITAAEFHPMSCNWFMYASSKGTIKLADMRESALCDQHAK---------------
AN1545.1  	EELTEVITAAEFHPTSCNWFMYASSKGTIKLADMRQRALCDTHHKRMCFSSLSSSRDTFE
          	************** *** ****************: **** * *  . :: ::: .: .

MG05637.1 	--------M1FEQEEDPSSRSFFSEIISSISDVRFSYDGRYVLSRDYLTVKIWDVNMEKQ
NCU09377.1	--------M1FEQEEDPSSRSFFSEIISSISDVRFSHDGRYILSRDYLTVKIWDVNMERQ
FG01369.1 	--------L1FEQEEDPSSRSFFSEIISSISDVRFSHDGRYILSRDYLTVKIWDINMERQ
AN1545.1  	SSSNLPYVV~FEQEEDASSRSFFSEIISSISDVRFSHDGRYIVSRDYLTVKIWDVNMERQ
          	:::. .  : ******.*******************:****::***********:***:*

MG05637.1 	PVKTIPIHEHLRPRLCDTYENDSIFDKFEVVFSGDAKNVMTGSYNNNFMIYPSDPEKETE
NCU09377.1	PVKTIPIHEHLRPRLCDTYENDSIFDKFEVVFSGDAKNVMTGSYNNNFMIYPSDPDKEVE
FG01369.1 	PVKTIPIHEHLRPRLCDTYENDSIFDKFEVVFSGDAKNVMTGSYNNNFMIYPSDPDKEVE
AN1545.1  	PVKTIPIHEHLRPRLCDTYENDSIFDKFEVVFSGDAENVMTGSYNNNFMIYPTDPAKETE
          	************************************:***************:** **.*

MG05637.1 	VVLQADKSAFKAKKVGVPTPINSSTSPTANGGKKGGSRAGSPAAGGQGQRMRKETDADQI
NCU09377.1	VVLQADKSAFKAKKVGVPTPINSSTSPTANGGKKGNSRAGSPAAAGQGQRMRKETDADQI
FG01369.1 	VVLQADKSAFKAKKVGVPTPINSSTSPTATNGKKGGSRAGSP--GGQGQRMRKETDADQI
AN1545.1  	IVLQADKSAFKAKKVGVPTPMN-----KGANGKKSNSRTSSP--AGPGSRMKKETDADQI
          	:*******************:*     .. .***..**:.**  .* *.**:********

MG05637.1 	DFNKKILHMSWHPFEDSIAIAATNN0AACNPVVGKSTRCKLTLFFLAQLFVFSAL
NCU09377.1	DFNKKILHMSWHPFEDSIAIAATNN0----------------------LFVFSAL
FG01369.1 	DFNKKILHMSWHPFEDSIAIAATNN0----------------------LFVFSAL
AN1545.1  	DFNKKILHMSWHPFEDSIAIAATNN0----------------------LFVFSAL
          	*************************                       *******
```
